# Research on Multi-Object Sorting System Based on Deep Learning

**DOI:** 10.3390/s21186238

**Published:** 2021-09-17

**Authors:** Hongyan Zhang, Huawei Liang, Tao Ni, Lingtao Huang, Jinsong Yang

**Affiliations:** 1 School of Mechanical and Aerospace Engineering, Jilin University, Changchun 130022, China; zhanghy@jlu.edu.cn (H.Z.); lianghw19@mails.jlu.edu.cn (H.L.); jsyang18@mails.jlu.edu.cn (J.Y.); 2 School of Vehicle and Energy, Yanshan University, Qinhuangdao 066004, China; nitao@jlu.edu.cn

**Keywords:** robot sorting, rotating target detection, instance segmentation, pose estimation

## Abstract

As a complex task, robot sorting has become a research hotspot. In order to enable robots to perform simple, efficient, stable and accurate sorting operations for stacked multi-objects in unstructured scenes, a robot multi-object sorting system is built in this paper. Firstly, the training model of rotating target detection is constructed, and the placement state of five common objects in unstructured scenes is collected as the training set for training. The trained model is used to obtain the position, rotation angle and category of the target object. Then, the instance segmentation model is constructed, and the same data set is made, and the instance segmentation network model is trained. Then, the optimized Mask R-CNN instance segmentation network is used to segment the object surface pixels, and the upper surface point cloud is extracted to calculate the normal vector. Then, the angle obtained by the normal vector of the upper surface and the rotation target detection network is fused with the normal vector to obtain the attitude of the object. At the same time, the grasping order is calculated according to the average depth of the surface. Finally, after the obtained object posture, category and grasping sequence are fused, the performance of the rotating target detection network, the instance segmentation network and the robot sorting system are tested on the established experimental platform. Based on this system, this paper carried out an experiment on the success rate of object capture in a single network and an integrated network. The experimental results show that the multi-object sorting system based on deep learning proposed in this paper can sort stacked objects efficiently, accurately and stably in unstructured scenes.

## 1. Introduction

The sorting scenes in current daily life, such as family services, garbage sorting and logistics distribution, are often unstructured scenes [1]. In the face of these environments, the robot system not only needs to identify and locate the target object but also needs to understand the whole environment. Many existing methods generally only identify and locate objects in the scene by target detection or template matching, which cannot play a stable sorting effect in the face of a variety of unknown object sorting scenarios with stacking. Because of the lack of understanding of the spatial relationship of objects in the scene, the target object is damaged in the process of grasping. Therefore, it is of great significance to study how to use robots for safe, stable and accurate sorting of complex multi-object scenes.

To solve the above problems, this paper proposes a multi-objective sorting system based on fusion neural network. In this system, we mainly proposed: (1) The attitude information of the object is obtained by two different networks so as to make full use of the advantages of the two networks. (2) Normal vector estimation based on point cloud processing and grasping sequence reasoning based on average depth calculation are proposed. The rotating target detection network is responsible for the position of the target object and the rotation angle on the horizontal plane. The instance segmentation network is responsible for obtaining the point cloud of the object surface. The point cloud data are used to calculate the normal vector of the upper surface and the average depth of each upper surface to calculate the grasping order. The normal vector is estimated by principal component analysis. In sorting order reasoning, we propose a new method. Firstly, each kind of object is placed on the experimental platform, and the variance of its average depth is recorded as a priori knowledge. Then, the mean value of the obtained point cloud data is sorted in the sorting process, and the grasping sequence is obtained by synthesizing the variance information of the prior knowledge. The system enables the robot to sort multiple unknown objects in the scene independently, stably and safely.

## 2. Rated Work

The sorting system is originally based on shallow learning. For example, K. Rahardja [2] and Ata AA [3] implement object recognition and pose estimation through edge detection technology, simple features of complex objects, color attributes and structural light principles, respectively. In addition, in the field of machine learning and robot system integration, D. Kragic and H. I. Christensen [4] use view-based support vector machine to realize the recognition and detection of daily household goods. In 2006, Ashutosh Saxena et al. [5] first used machine learning to predict the grasping position of daily necessities and realized the effective grasping of unknown objects. Wang [6] realized the classification prediction and location determination of sorting objects through SVM and circle projection features. The saliency detection algorithm is used to remove the background of the image, and the upper and lower relations of the stacked objects are judged by combining the depth information.

In recent years, compared with shallow learning, deep learning has great advantages in extracting and learning image features. Levine [7] et al. predicted a given action instruction by grasping the prediction network to calculate the possibility of grasping and then combined the servo mechanism with the neural network to continuously select the best path to achieve successful grasping. D. Hossain [8] et al. constructed a system that can identify and select targets in different positions, directions and illuminations by combining DBNN network with evolutionary algorithm. In addition, Ulrich Viereck et al. [9] built a stacked object sorting system based on convolutional neural network with learning distance function. Zhang Xinyi [10] realized the automatic sorting system of stacked multi-objects by combining semantic segmentation, three-dimensional reconstruction and support relationship reasoning. Li [11] realized the recognition of the position and category of multi-objects with uncertain positions in the environment and drove the robot to grab and sort. On this basis, Wang Bin [12] added the method of visual and force fusion to complete the compliant control of the robot in the sorting process. However, the above methods are only applicable to the separated multi-object sorting scene. When objects in the scene are randomly stacked, these methods may cause object damage or grab failure during sorting.

At present, the rotating frame detection method is mainly divided into single-stage, two-stage and three-stage network. The single-stage network runs faster, but the accuracy is low. The multi-stage network has good performance, but the speed is slow, and the construction is difficult. The representative of single-stage network is R3Det [13], while the main representative of dual-stage network is R2CNN [14]. In the three-stage network, Jian Ding [15] proposed a three-stage rotating target detection architecture based on ROI Transformer module. ROI Transformer module is a common module, which can be added to other frameworks to improve the detection accuracy. Since the rotation angle of rotating rectangular border is not easy to learn, Yongchao Xu et al. [16] proposed a Gliding vertex network architecture that can predict quadrilateral borders. The network uses the offset of four corners relative to the horizontal rectangular border to describe the quadrilateral bounding box of the object. RSDet [17] also uses four corners to represent the rotating frame of the object. For the boundary problem, RSDet solves it by moving the ordered corners forward or backward and then calculating the minimum loss.

As for the case segmentation network, it is mainly divided into three categories: (1) the top-down detection-based model, (2) a bottom-up model based on semantic segmentation, and (3) the direct instance segmentation model. However, for objects with complex or large contours, most networks have rough segmentation of their boundaries. 

Yi Li [18] constructed a highly integrated and efficient end-to-end network FCIS by expanding the FCN [19] method, which can jointly solve the problem of target classification and instance segmentation. Xiaoke Shen [20] proposed A 3D convolutional-based system, which generates frustums from 2D detection results, proposes 3D candidate voxelized images for each frustum and uses a 3D convolutional neural network (CNN) based on these candidates voxelized images to perform the 3D instance segmentation and object detection. He Kaiming [21] et al. proposed the classic instance segmentation network Mask R-CNN in 2017. The algorithm realizes the segmentation of objects in the scene by adding mask head network to the target detection network Faster R-CNN. This method also innovatively changes the original ROI pooling to ROI Align, which ensures the feature alignment from the original image to ROI, thereby improving the performance of the entire network. Daniel Bolya et al. [22] proposed the real-time instance segmentation network model YOLACT for the first time. YOLACT innovatively changed the original NMS strategy to Fast NMS, thus maintaining the accuracy of the model and improving the speed of non-maximum suppression operation. Sida Peng [23] improved snake method and proposed a bottom-up DeepSnake network architecture. DeepSnake also has good segmentation speed and accuracy. Wang Xinlong et al. [24] proposed a network architecture SOLO, which is different from the previous design ideas. The network innovation transforms the instance segmentation problem into the instance type problem of the prediction pixel, and the overall network architecture is very simple. The SOLO network has both high speed and segmentation accuracy. However, since SOLO distinguishes different instances through the location information of objects, the network has poor effect when the objects are stacked.

In terms of the representation method of grasping pose, in recent years, $\left\{x,y,w,h,\theta \right\}$ are mostly used to represent the grasping pose of the object [25,26,27]. For a multi-object sorting scene, when the object is stacked and the stacked object has a certain height, the use of five parameters to guide the robot grasping can easily lead to grasping failure or even damage to the target object. Therefore, this paper uses $\left\{x,y,w,h,\theta ,\varphi \right\}$ to represent the grasping pose. The rotating rectangular frame is defined by the long edge method, where (x,y) represents the center point coordinates, the long edge of the rectangular boundary frame is defined as h, and the other edge is defined as w. $\theta \in \left[-90^{\circ },90^{\circ }\right]$ is the angle of the long side h and x axis positive direction, and the increase of φ is used to represent the angle between the normal vector of the object surface and the angle perpendicular to the horizontal direction. x,y,w,h and θ are obtained by rotating target detection network, and parameter φ is obtained by instance segmentation network.

## 3. Introduction of Experimental System

### 3.1. Experimental Platform of Sorting System

**Figure 1:**
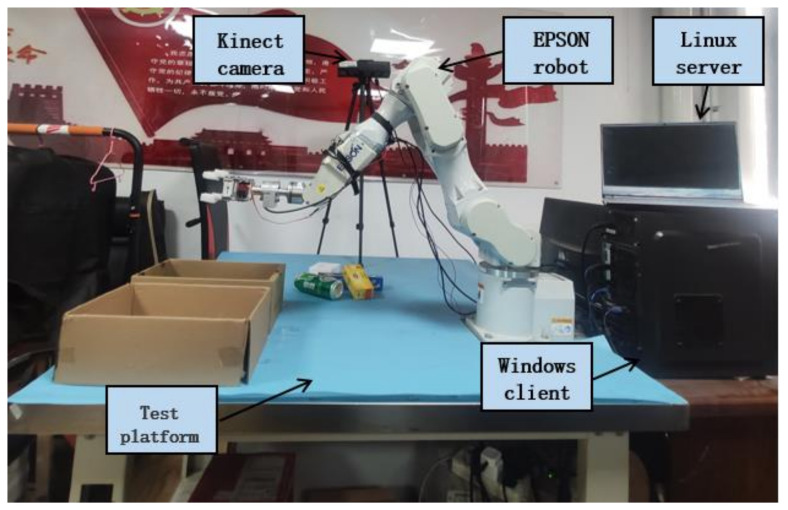
Sorting experimental platform.

The experimental platform of sorting system is shown in Figure 1, including: EPSON C4-A901 robot, Kinect camera, Linux server and Windows client. The EPSON robot is placed on one side of the test bench, and the Kinect camera is fixed on the other side. The Kinect camera is elevated to a distance of 1 m from the desktop using a tripod and adjusted to a 75 °angle of view. The configuration between the camera and the robot in this paper belongs to eye-to-hand, that is, the camera is installed in the external fixed position of the robot. In this case, the robot–camera system is linked by four closed chains of Euclidean transformations. In this paper, the Zhang Zhengyou calibration method is used to calibrate the collected images with good exposure so as to obtain the internal and external reference matrix [28,29,30] of the camera.

Kinect camera is connected to the Linux server through the USB line so as to transmit the collected information to the Linux server. The Linux server is equipped with a 6G GTX 1660 Ti display card for running algorithms such as rotating target detection, instance segmentation, normal vector estimation and sorting order reasoning so as to process visual and depth information collected by Kinect. The communication between Linux server and Windows client is based on TCP/IP protocol. The Windows client will receive the information and convert it to the control instructions of the robot. The robot controller controls the robot to complete the sorting task. Cookie box, tea box, mouse box, beer can and apple are employed as experimental objects.

### 3.2. Software Design of Sorting System

**Figure 2:**
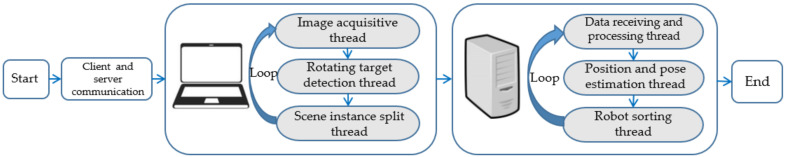
Operation process of robot sorting system software.

The running process of robot sorting system software is shown in Figure 2. Firstly, the experimenters complete the communication between the two ends by running the server program and the client program respectively. Then the server runs the image acquisition thread, the rotation target detection thread and the scene instance segmentation thread in sequence. The image acquisition thread uses Kinect camera to obtain color images and depth images from the scene and saves the image information. The rotating target detection thread predicts the collected color images through the R3Det network to obtain the information of the target object category, position coordinates and rotation angle in the scene. The scene instance segmentation thread includes instance segmentation, point cloud processing, normal vector estimation and sorting order reasoning algorithm. The sorting order and surface normal vector of the target object can be analyzed by using the input image information.

The data receiving and processing thread of the client receives and processes the target object information transmitted by the server and saves the information. After the pose estimation thread estimates the pose of the object, the robot sorting thread performs the grasping operation according to the sorting order so as to complete the sorting task.

## 4. Rotating Target Detection Network

### 4.1. Basic Framework

**Figure 3:**
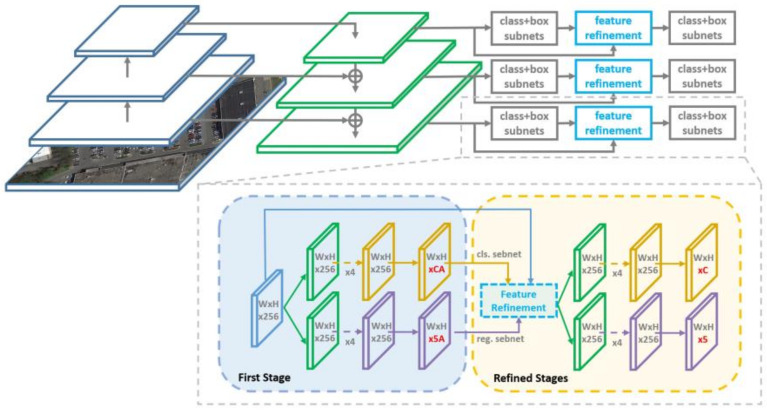
R3Det Overall network framework.

The overall frame of R3Det [31] network is shown in Figure 3. R3Det network is divided into three parts:

(1) Basic network RetinaNet: RetinaNet uses ResNet(Residual Network) + FPN (Feature Pyramid Networks) as the backbone network and uses single-stage target detection and Focal Loss. RetinaNet is used to realize the preliminary detection of rotating targets in the first stage.

(2) Feature alignment module: By reconstructing the whole feature map on the feature points, the problem of unalignment of cascade structure features is solved to obtain more accurate boundary position information.

(3) Refining module: cascade optimization is adopted to improve the accuracy of classification and the positioning accuracy of regression boundary by cascading refining modules (multiple can be added) after the first stage.

### 4.2. Dataset Construction

In this paper, the rotating target detection is applied to the robot grasping task, and the targets in the existing public data set (COCO/DOTA) do not meet the grasping requirements of the robot system. Therefore, the data set needs to be constructed according to the types of robots and manipulator claws.

The constructed dataset needs to meet the following conditions;

(1) In order to ensure that the trained neural network has good effect and good generalization, the type and number of target objects selected to construct the data set should be as many as possible and able to be captured by the existing robot paws.

(2) Image resolution should be consistent with the resolution of the image taken in practical application. In this paper, KinectV2 camera is used to obtain the image of the scene in the sorting process, so when collecting the data set, KinectV2 is also used to collect images with resolution of 1920 × 1080.

(3) In order to achieve the training of the rotating target detection model, not only should the category and position of the object be marked, but the rotation angle of the target should also be marked when labeling the image.

**Figure 4:**
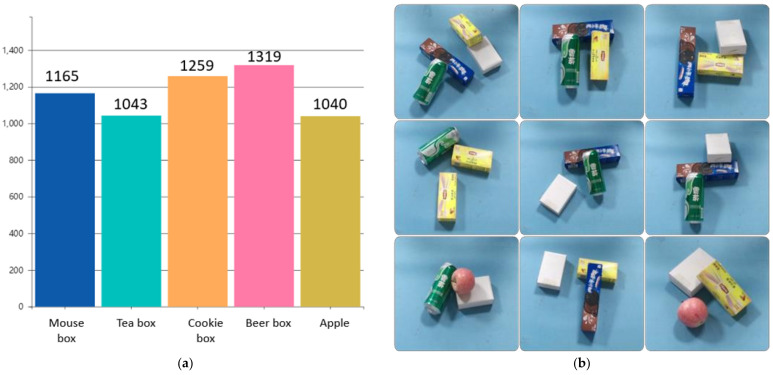
Data set object number distribution (**a**) and data set image examples (**b**).

According to the above conditions, five kinds of objects suitable for grasping are selected as the target objects in the construction of data sets. When collecting images, 2~4 target objects are placed on the experimental platform. Due to the random placement of objects in practical application scenarios, objects may be stacked. Therefore, the collected image contains a variety of situations, such as separate objects, multiple objects scattered and multiple objects containing stacking. Finally, a total of 2000 images of target objects in the experimental scene were collected, with a total of 5826 entity instances. The number distribution of dataset objects and dataset images are shown in Figure 4.

**Figure 5:**
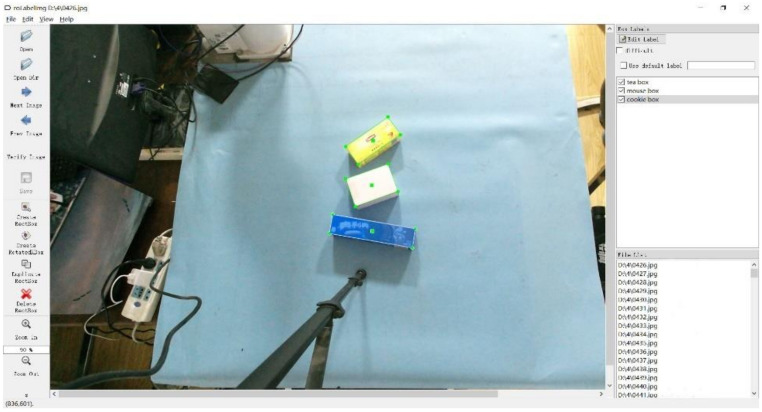
Labeling process.

Calibration of data sets refer to PASCAL VOC [32] including position information of real labels, specifications and posture of objects (rotation angle of marked border *θ*). RoLabelImg is the dataset tagging software we use. The specific annotation process is as follows: Firstly, draw the horizontal border, which is similar to the size of the object in the image. Then, rotate the horizontal frame to make its center point and edge coincide with the target object center point and the upper surface contour, respectively. Finally, add the object category label to the rotating border, as shown in Figure 5. After labeling, the dataset is divided into a training set with 1600 images and a test set with 400 images, and then the Python program is used to change the annotation format to the DOTA dataset format (four corner coordinates of the rotating border) for training the neural network model.

### 4.3. Model Training and Evaluation

#### 4.3.1. Dataset Augmentation

**Figure 6:**
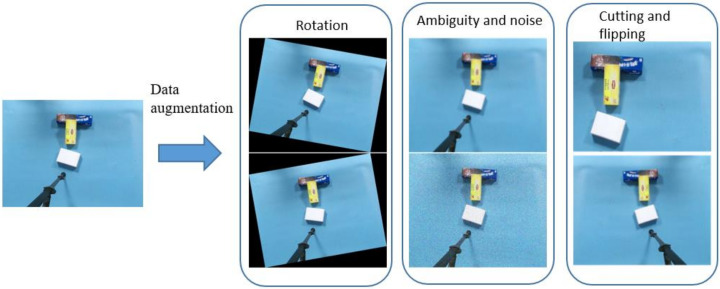
Data enhancement transformation effect diagram.

The size of the data set plays a vital role in achieving good performance of neural network model training. Data set enhancement is a method to generate data equivalent to the original data set through a series of transformations. Thus, the target network can be applied in various scenarios, such as different shooting direction, location and illumination when collecting data. In this paper, the original data set is enhanced by horizontal reversal, rotation, clipping, blurring and Gaussian noise, as shown in Figure 6. For each image, random transformation is carried out according to the probability set by each transformation, and the transformation method can be superimposed. Therefore, the new data generated may contain multiple transformations.

#### 4.3.2. Model Training

This paper constructs a rotating target detection model based on Ubuntu18.04 + tensorflow deep learning framework. The hardware configuration is Intel (R) Core (TM) i7-10700F CPU, 16GB memory and 8GB Nvidia GeForce RTX 2070 SUPER display card.

In model training, momentum optimizer is applied, and momentum is set to 0.9. The number of images sent to the model for each step of training is 1. For each input image, the random data enhancement method described in Section 4.3.1 is adopted. In this paper, the learning rate adopts the strategy of warming up first and then decaying. The learning rate changes in the process of gradually increasing first, then stabilizing and finally decreasing in segments.

**Figure 7:**
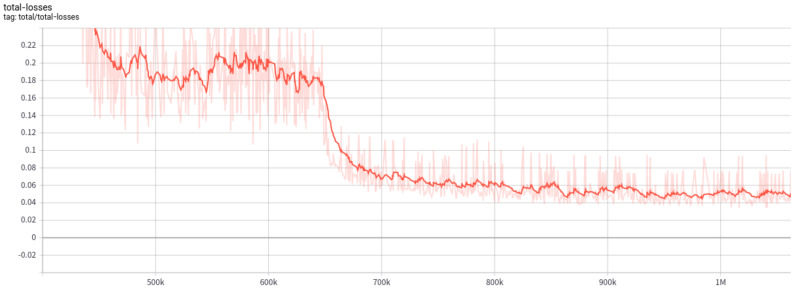
Curve diagram of overall training loss.

Neural network training is monitored by Tensorboard, and the overall training loss is shown in Figure 7. It can be seen from the figure that the loss gradually decreases at the initial stage and then tends to be stable, and the ladder-like decline occurs at the global step number of about $6.5\times 10^{5}$ steps, which is caused by the segmented decrease of learning rate. In a certain stage of neural network training, if the learning rate is large, it will lead to the model crossing the local optimal solution. When the learning rate decays, the loss continues to decline, and the model finally converges.

#### 4.3.3. Model Evaluation

First, the IoU (Intersection over Union) threshold of the rotating target detection model is set to 0.5.

The prediction results of statistical samples, recall and precision sometimes appear contradictory situation; one of them is very high, and the other is very low. Therefore, it is necessary to adopt the F score for comprehensive consideration, whose expression is

(1) $$F=\frac{\left(\alpha^{2}+1\right)PR}{\alpha^{2}\cdot \left(P+R\right)}$$

Among them, *P* represents precision and *R* represents recall. α is a constant, usually take 1. F-Score combines recall and precision: the higher the value, the better the performance of neural network.

**Figure 8:**
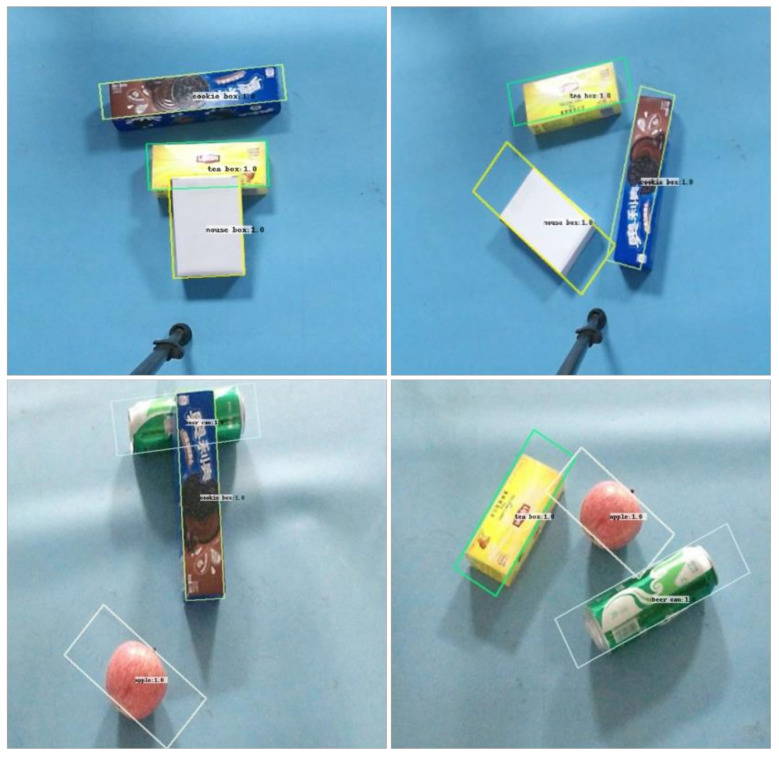
Detection effect of rotating target detection network.

Table 1 shows that the F-Score of the rotating target detection network model constructed in this paper is 0.99%. Compared with the conventional rotating target monitoring network [33], the detection performance of the network model is good, and part of the test set after neural network detection is shown in Figure 8.

## 5. Instance Segmentation Network

### 5.1. Basic Framework

**Figure 9:**
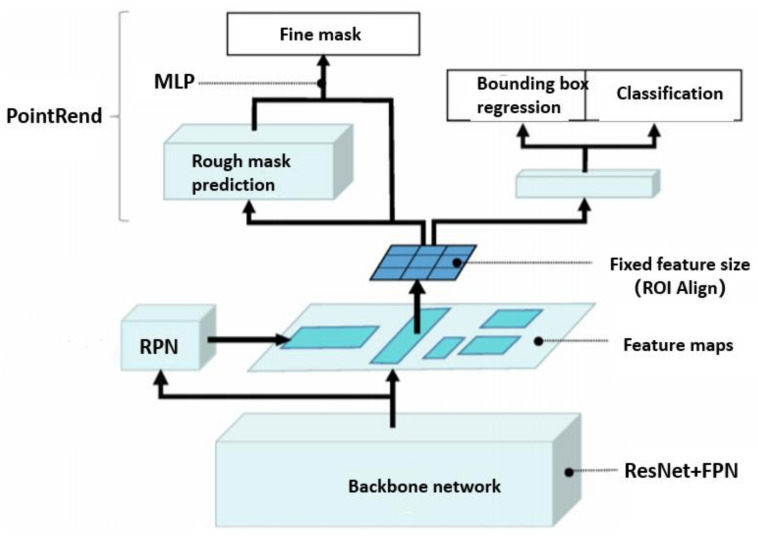
Optimizing the overall structure of Mask R-CNN.

The overall framework of the optimized Mask R-CNN model is shown in Figure 9. The basic framework is mainly divided into backbone network (ResNet + FPN), regional proposal network (RPN), region of interest alignment module (ROIAlign), classification branch, regression branch and mask branch (Point Rend). Similar to Faster R-CNN, the optimized Mask R-CNN is also a two-stage network model. The main workflow is as follows: Firstly, the input image is processed by the feature extraction network composed of ResNet and FPN, and the multi-scale feature map is generated as the input of the subsequent network. Then the anchor of the corresponding scale is fixed on each feature map to generate the ROI. The RPN network makes a preliminary classification and border regression of the input ROI and filters ROI to generate region proposal. Then, the generated region proposal is input to ROI Align to align the original graph with the feature graph and convert the feature graph to the same size. Then, it is divided into two routes. One route predicts the category and location of ROI, and the other Point Rend branch predicts the mask of the input ROI and finally obtains the high-resolution mask.

### 5.2. Dataset Construction and Pre-Processing

**Figure 10:**
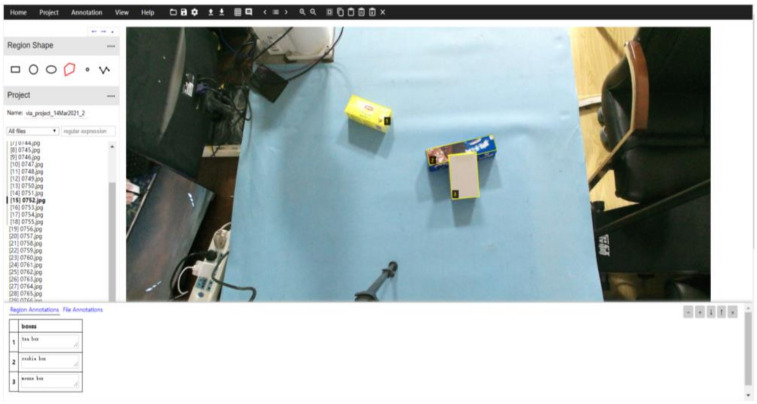
VIA Labeling interface.

The data of instance segmentation dataset still uses 2000 images collected in 4.2 as data and randomly divides them into instance segmentation training set and test set according to the 10-Fold Cross Validation. The training set and test set are marked by polygons through VIA annotation tool, and the segmented labels are saved as. json format files. The annotation schematic is shown in Figure 10.

After the data set annotation is completed, the Python program is written to convert the format of the .json file so as to obtain the segmentation label suitable for optimizing the Mask R-CNN model training.

### 5.3. Model Training

This paper constructs an instance segmentation model under the framework of Ubuntu 18.04 + Detectron2 deep learning, and the hardware configuration is the same as before. The model uses ResNet50 + FPN as feature extraction network to obtain multi-scale features from input. During the model training, the momentum SGD optimizer is used for optimization. The momentum value is 0.9, and the weight attenuation of 0.0001 is applied. Similarly, the pre-heating training strategy is adopted. The initial learning rate is set to 0.002, and the pre-heating coefficient is 0.001. The learning rate of the model begins to decrease at a rate of 0.1 after 7000 iterations. For instance, partition training sets train 10 epochs and set the batch size for each step to 2. During training, the data set is also enhanced by means of horizontal turnover and scale change.

### 5.4. Model Evaluation

This paper takes Mean IoU (MIoU) as the standard of evaluation.

Assuming that the *K* + 1 class (including background) target object training model is used, $p_{ij}$ is used to represent the number of pixels that are actually labeled as Class i but are predicted as Class j. When i is a positive class and i is not equal to j, $p_{ii}$ represents the true positive case (*TP*), and $p_{ij}$ and $p_{ji}$ represent false positive case (*FP*) and false negative case (*FN*), respectively. At this point, the merge ratio can be expressed as

(2) $$IoU=\frac{TP}{TP+FP+FN}$$

The average merge ratio is the average value of the merge ratio for all classes. The formula is

(3) $$MIoU=\frac{1}{K+1}\sum_{i=0}^{K}\frac{p_{ii}}{\sum_{j}^{k}p_{ij}+\sum_{j}^{k}p_{ji}-p_{ii}}$$

According to the above indicators, the test data set is used for evaluation. All IoUs of each type of object in the data set are obtained through experiments, and the average value is used to reduce the error. Finally, the MIoU of the instance segmentation model is obtained by averaging the IoU of all classes, and the results are shown in Table 2.

**Figure 11:**
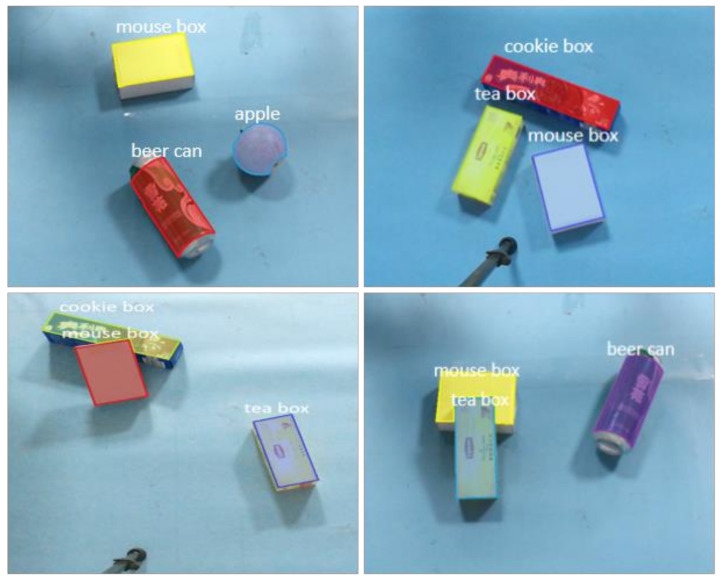
Instance segmentation effect.

Table 2 shows that the MIoU value is 73.52%. Since MIoU is about 70% of the common instance segmentation results, the optimized Mask R-CNN constructed in this paper has a good segmentation effect. In fact, the results of segmentation visualization are shown in Figure 11.

## 6. Attitude Estimation and Sorting Order Reasoning

### 6.1. Normal Line Estimation of Object Surface Based on PCA

The instance segmentation network can segment the object from the scene to obtain the pixel coordinates of the object surface. The pixel coordinates of the object surface are converted into point clouds in the world coordinate system by the internal and external reference matrix obtained by camera calibration. The world coordinate corresponding to the *i* pixel is $p_{i}\;$*=* ($x_{i},y_{i},z_{i})$. Point cloud is a set of *n* points, which is expressed as $P=\left\{p_{1},p_{2},\cdots ,p_{n}\right\}$.

#### 6.1.1. Point Cloud Down-Sampling

**Figure 12:**
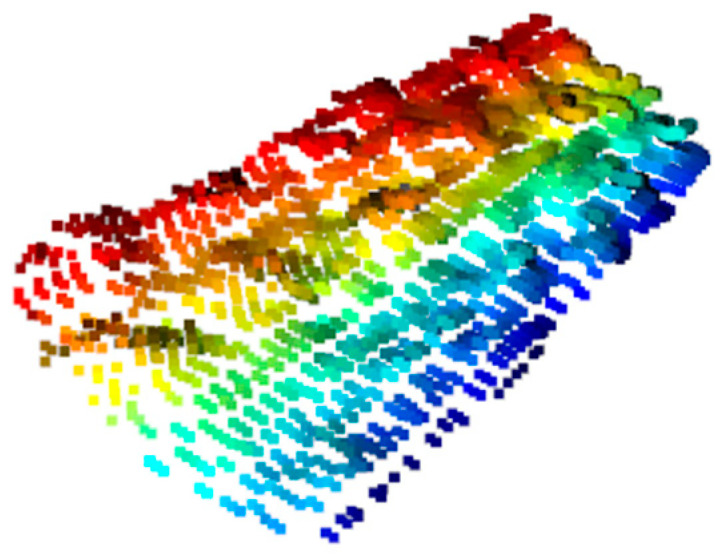
Cloud down-sampling on tea box surface.

In order to improve the processing speed of point cloud and maintain its inherent geometric characteristics at the meantime, the point cloud is processed by down-sampling so that the number of points in the point cloud is reduced from 10,000 to about 3000. The point cloud after down-sampling is shown in Figure 12.

Figure 12 shows the point cloud on the surface of the biscuit box, and the color does not mean anything. It can be seen that the overall distribution of the upper surface of the object (cookie box) is the same as that of the actual object (cookie box) after point cloud sampling. The subsequent experiments show that point cloud down-sampling has little effect on the error of the normal vector of the upper surface of the object and can still ensure the success rate of grasping.

#### 6.1.2. Point Cloud Principal Component Analysis

**Figure 13:**
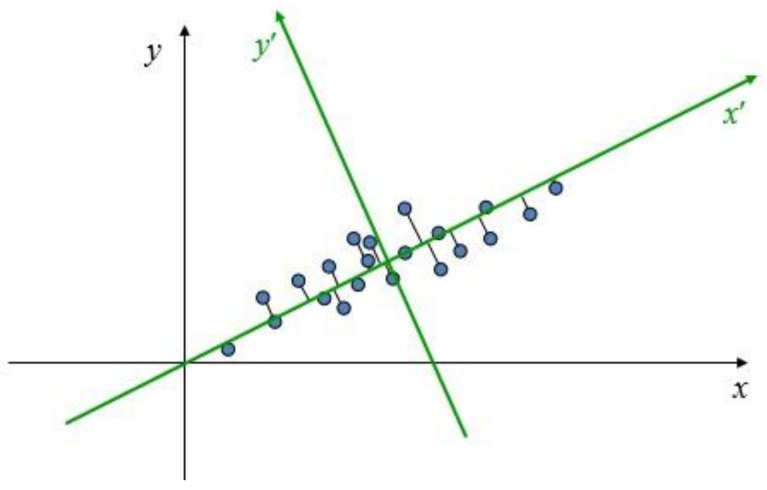
PCA theory.

Normal line estimation of object surface based on PCA is to analyze the main change direction of point cloud by projecting point cloud to a plane, as shown in Figure 13.

PCA first calculates the average position of input point cloud data. The formula is

(4) $$\bar{p}=\frac{1}{n}\sum_{i=1}^{n}p_{i}$$

In order to eliminate the influence of the value of point cloud coordinates, the point cloud data are de-meaned. The normalized zero-mean point cloud data are denoted as $\tilde{P}=\left\{{\tilde{p}}_{1},{\tilde{p}}_{2},\cdots ,{\tilde{p}}_{n}\right\},{\tilde{\;p}}_{i}=p_{i}-\bar{p}$. Then, a covariance matrix *M* is constructed as follows:

(5) $$M=\frac{1}{n}\overset{n}{\underset{i=1}{\sum }}{\tilde{p}}_{i}{\tilde{p}}_{i}{}^{T}$$

The covariance matrix is a symmetric matrix, where the diagonal elements represent the variance of the x, y and z coordinates of the point cloud, rather than the diagonal elements describing the correlation between the x, y and z coordinates of the point cloud. The elements in matrix *M* can be represented as

(6) $$\begin{array}{c} M_{11}=\frac{1}{n}\overset{n}{\underset{i=1}{\sum }}{\left(x_{i}-{\bar{p}}_{x}\right)}^{2},\;M_{12}=M_{21}=\frac{1}{n}\overset{n}{\underset{i=1}{\sum }}\left(x_{i}-{\bar{p}}_{x}\right)\left(y_{i}-{\bar{p}}_{y}\right)\\ M_{22}=\frac{1}{n}\overset{n}{\underset{i=1}{\sum }}{\left(y_{i}-{\bar{p}}_{y}\right)}^{2},\;M_{13}=M_{31}=\frac{1}{n}\overset{n}{\underset{i=1}{\sum }}\left(x_{i}-{\bar{p}}_{x}\right)\left(z_{i}-{\bar{p}}_{z}\right)\\ M_{33}=\frac{1}{n}\sum_{i=1}^{n}{\left(z_{i}-{\bar{p}}_{z}\right)}^{2},\;M_{23}=M_{32}=\frac{1}{n}\sum_{i=1}^{n}\left(y_{i}-{\bar{p}}_{y}\right)\left(z_{i}-{\bar{p}}_{z}\right) \end{array}$$

SVD operation is performed on matrix *M* to obtain its eigenvalues $\lambda_{1}$, $\lambda_{2}\;$and $\lambda_{3}$. Take the eigenvalues back to matrix *M* to calculate the corresponding eigenvectors $V_{1},V_{2}$ and $V_{3}$. The physical meaning of covariance matrix is the correlation of point clouds along specific direction *V*. The smaller the eigenvalues of matrix *M*, the smaller the correlation of point cloud in the direction of feature vector calculated by the eigenvalues. Since the point cloud of the object surface has little correlation in the normal vector direction, the feature vector corresponding to the minimum values in $\lambda_{1},\lambda_{2}$ and $\lambda_{3}$ is the estimation of the normal line of the object surface.

**Figure 14:**
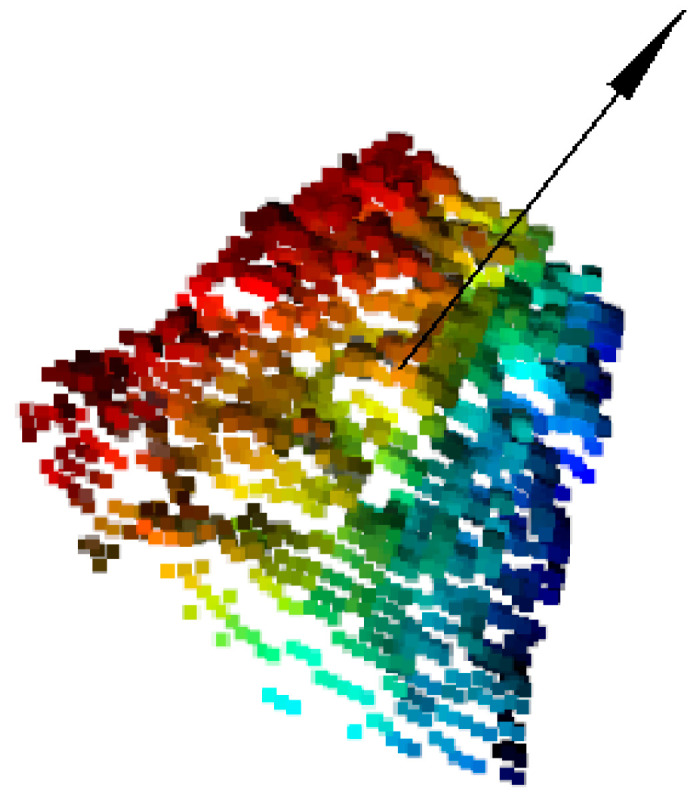
Normal line estimation results of tea box surface.

However, the calculated normal vector has two directions, so it needs to be redirected according to its relationship with the positive direction of *z* axis in the world coordinate system:

(7) $$n=\left\{\begin{array}{c} n,\\ -n, \end{array}\right.\begin{array}{c} n\cdot z>0\\ else \end{array}$$

where *n* represents the normal of the object surface, and *z* represents the positive direction of *z* axis in the world coordinate system. The normal estimation results of the object surface are shown in Figure 14.

### 6.2. Three-Dimensional Attitude Estimation of Target Based on Euler Angle Method

**Figure 15:**
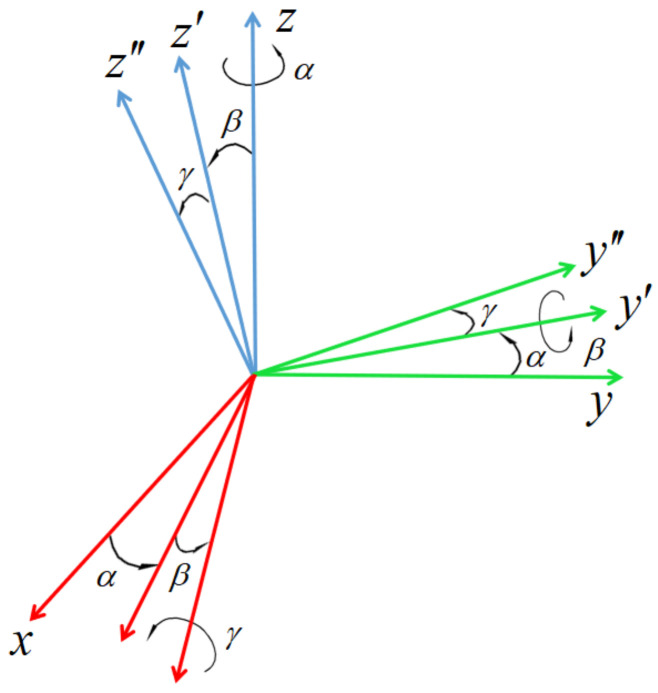
Eulerian angle.

The representation of the grasping pose represents the information needed for the pose estimation of the object. In this paper, the position information $\left(x,y\right)$ of the target object and the rotation angle θ around the *z* axis of the world coordinate system (coincident with the robot base coordinate) are obtained by the rotating target detection model. Then, in this section, the object is segmented by the instance segmentation model, and the normal vector of the object surface is obtained by the principal component analysis technology. By calculating the *z* angle between the normal vector of the object surface and the positive direction of the *z* axis of the world coordinate system, the rotation angle φ of the object around the *y* axis of its own coordinate system can be obtained. The position of the target object in the robot base coordinate system can be solved by external reference matrix obtained after calibration, and the three-dimensional attitude is described by the Euler angle method, as shown in Figure 15.

As shown in Figure 15, the rotation of the coordinate axis in the Euler angle description method is carried out relative to a coordinate axis in the moving coordinate system. In this paper, according to the actual situation, the order of *z* axis, *y* axis and *x* axis is described. In the initial state, the *x*, *y* and *z* axis of the target object coordinate system coincide with the robot base coordinate system. Firstly, the target object coordinate system rotates angle α around its *z* axis, then rotates angle β around its y axis, and finally rotates angle γ around its *x* axis to obtain the final attitude. Its posture can be expressed as

(8) $$\begin{array}{c} R_{zyx}\left(\alpha ,\beta ,\gamma \right)=R\left(z,\alpha \right)R\left(y,\beta \right)R\left(x,\gamma \right)\\ =\left[\begin{array}{ccc} c\alpha c\beta & c\alpha s\beta s\gamma -s\alpha c\gamma & c\alpha s\beta c\gamma +s\alpha s\gamma \\ s\alpha c\beta & s\alpha s\beta s\gamma +c\alpha c\gamma & s\alpha s\beta c\gamma -c\alpha s\gamma \\ -s\beta & c\beta s\gamma & c\beta c\gamma \end{array}\right] \end{array}$$

where sα and cβ represent sinα and cosβ, respectively, and other elements in the matrix are similar.

According to the definition of Euler angle, it can be seen that the rotation angle θ corresponds to angle α, and the angle φ between the normal vector of the object surface and the positive direction of the *z* axis corresponds to angle β. Since the object is placed on the horizontal desktop in the sorting scene and the angle is very small even if there is stacking, the angle γ is ignored. Thus, the attitude of the target object in the world coordinate system is obtained.

### 6.3. Sorting Order Reasoning

For the randomly placed multi-object sorting scene, in order to realize the understanding and judgment of the scene and enable the robot to sort the target the target object in an autonomous, stable and safe manner, it is particularly critical to infer the grasping order of the object. The sorting task by reasonable grasping sequence can effectively avoid the damage of the target object due to falling or collision with the claw. People are very good at judging the relationship between the front and back of the object, generally through the eyes to obtain the depth of the object or according to the size of the object and occlusion characteristics of experience to determine the spatial semantic information between objects. In order to realize the sorting order reasoning function, based on the point cloud on the object surface segmented by the instance segmentation network, this paper judges the sorting order of objects in the scene according to the change characteristics of point cloud in the *z* axis direction.

In this paper, we first obtain the threshold of the variance of the point cloud of the object surface in the *z* axis direction without stacking through experiments. The specific method is as follows: Firstly, the *k*-th target object is placed at any position on the experimental platform, and the variance of the point cloud of the object surface in the *z* axis direction is calculated. Then, we repeat the above steps to obtain the maximum point cloud variance of class *k* target surface $\delta_{max}$. Finally, a value slightly greater than $\delta_{max}$ is selected as the threshold $\delta_{k}$ of point cloud variance on the surface of the *k*-type target object. Through experiments, the thresholds of five kinds of target objects are obtained as shown in Table 3.

**Figure 16:**
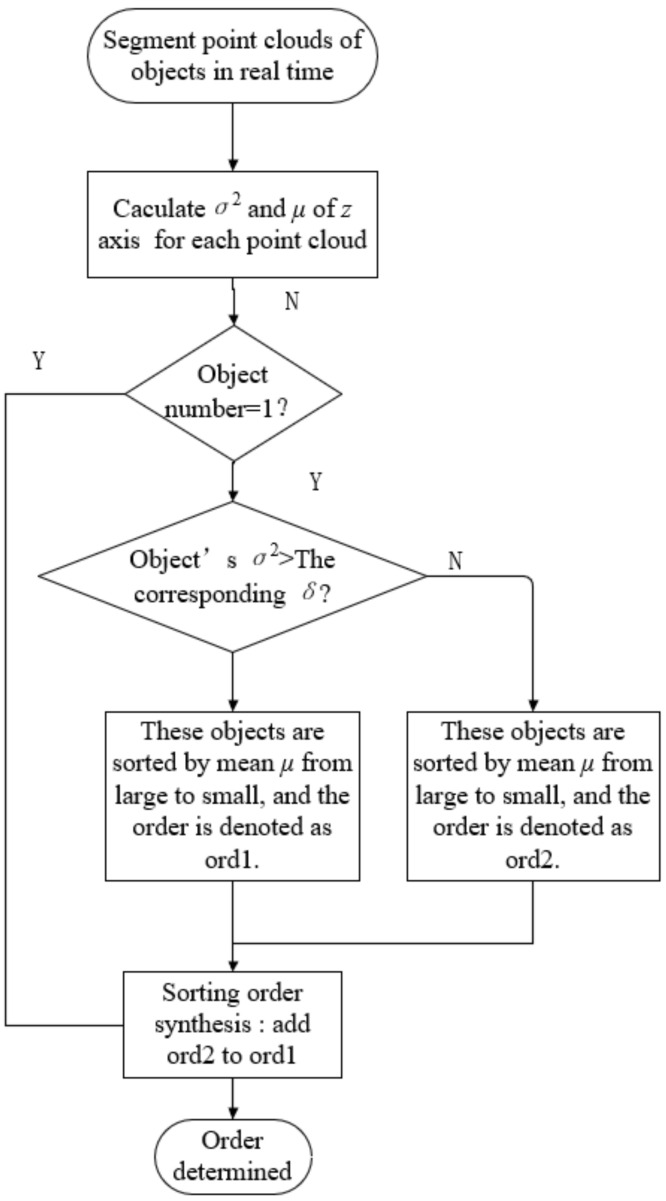
The sorting sequence reasoning algorithm flow.

The obtained threshold of the variance of the point cloud on the object surface is used as a priori knowledge to judge the stacking of objects in the scene. In the multi-object sorting task, through the calculation of the real-time segmentation of the object surface point cloud, the variance $\sigma_{k}{}^{2}$ and mean $\mu_{k}$ in the *z* axis direction are obtained. Then the size between variance $\sigma_{k}{}^{2}$ and threshold $\delta_{k}$ is compared, and the reasonable sorting order is deduced by combining mean $\mu_{k}$. The specific algorithm process is shown in Figure 16.

## 7. Experiment

### 7.1. Experimental Results of Rotating Target Detection and Instance Segmentation

#### 7.1.1. Detection Effect of Rotating Target

**Figure 17:**
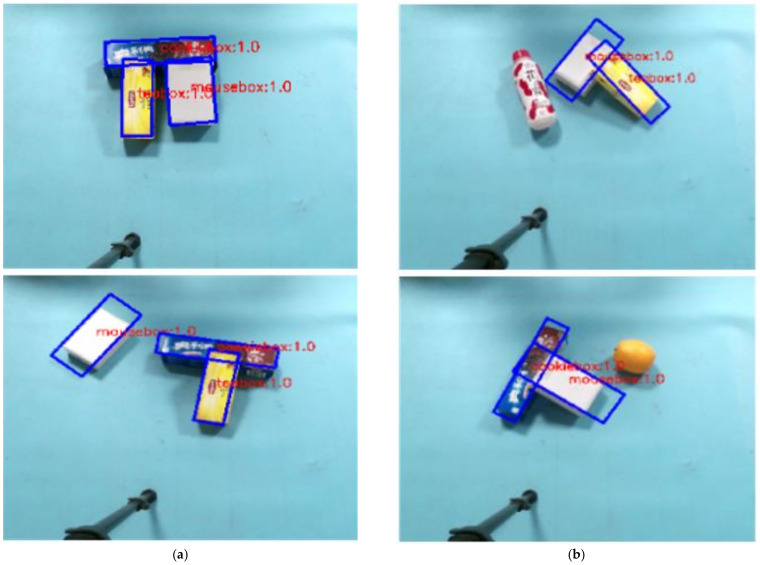
Prediction effect of rotating target detection network in actual scene. (**a**) The training set object in the scene; (**b**) the scene containing untrained objects.

In order to test the actual effect of rotating target detection network, 2~4 sorting objects are randomly selected and placed on the experimental platform, and the relationship between objects is stacked and separated. The real-time detection of target objects in the scene is realized by running the rotating target detection network on the server host alone. The experiment is conducted 50 times. Some results of the experiment are shown in Figure 17a. In the figure, the blue border is the rotation border predicted by the model, and the red font is the predicted category and confidence. In order to further detect the robustness of the network, the untrained objects and the target objects are placed on the experimental platform at the same time. It is also conducted 50 times. The part of the detection effect is shown in Figure 17b. Based on the above detection results, it can be seen that the rotation target detection model constructed in this paper can accurately identify the category of objects, and the predicted boundary frame position and rotation angle error are small, and the noise is less.

#### 7.1.2. Scene Instance Segmentation Effect

**Figure 18:**
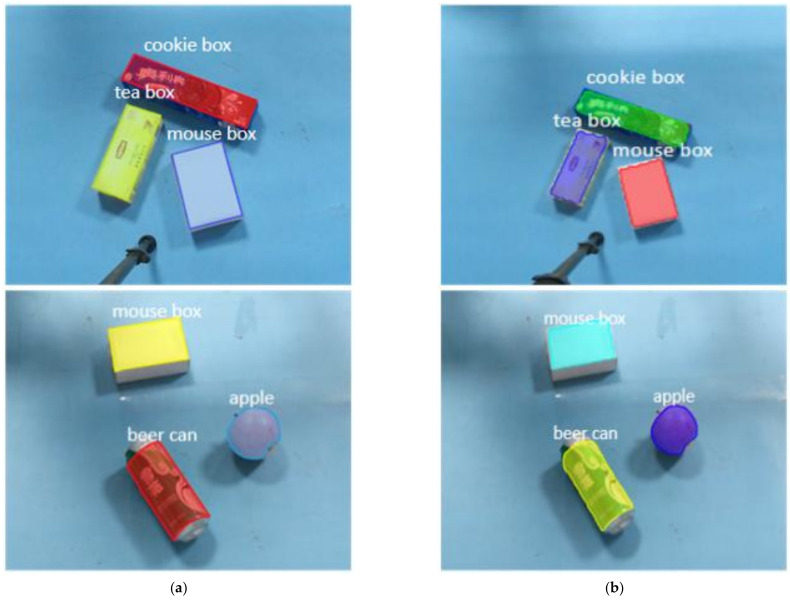
The actual scene instance segmentation network segmentation effect (**a**) for network detection results and (**b**) for unoptimized network detection results.

In order to test the effect of the instance segmentation model, 2~4 sorting objects are randomly selected and placed on the experimental platform, and the relationship between objects is stacked and separated. The real-time segmentation of the target object in the scene is realized by running the instance segmentation network on the server host separately. Each target instance in the mask with different colors is used in the experiment, and the segmentation results are shown in Figure 18a. This paper optimizes the model by replacing the mask branch of Mask R-CNN. In order to verify the optimization effect, the unoptimized network is used for experiments in the same scene, and the generated segmentation image is shown in Figure 18b. Compared with (a) and (b), we can see that the segmentation boundary in optimized Mask R-CNN (a) is smoother, and the optimization effect is obvious.

### 7.2. Robot Sorting Experiment

#### 7.2.1. Single Object Grasping Experiment

**Figure 19:**
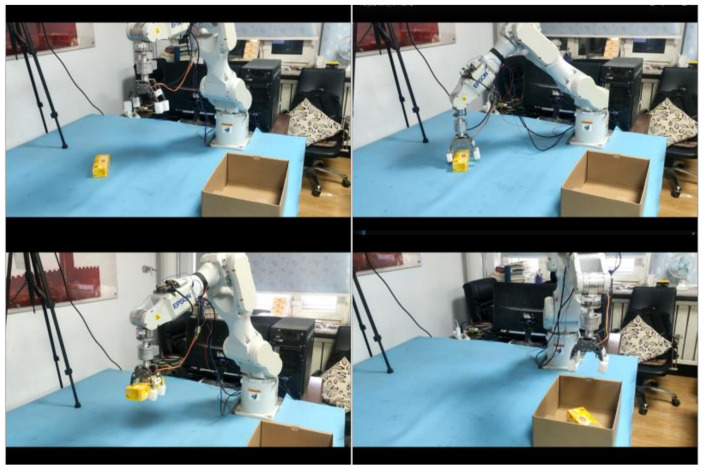
Single object grasping process.

In order to verify the grasping performance of the robot system, this group of experiments carried out 30 times of grasping for five categories of objects and ensured that the objects were placed at any posture and position during each grasping. The specific grasping process is shown in Figure 19. First, place a single object on the desktop. Then turn on the Linux server thread. The camera starts to obtain the image information and transmits the calculated object position information to the Windows client. After receiving the transmitted data, the operator clicks the grab button in the Windows client program. The lower computer starts to drive the robot to the top of the object according to the calculated object position coordinates. Then the claw posture is adjusted to the calculated posture. Then the claw gets close to the object. After a second delay, the manipulator claws will be raised and moved to the pre-positioned carton to complete the sorting task.

The experimental results are shown in the following Table 4.

The experiment carried out a total of 150 grabs for each of the five categories of items 200 times. From the grab results, it can be seen that the robot sorting system constructed in this paper has a high success rate of grasping regular objects such as boxes and a relatively low success rate of grasping irregular objects. The reason is that the constructed neural network has poor recognition effect on irregular objects, resulting in a certain deviation between the estimated grasping pose and the actual pose. However, in general, the method proposed in this paper has a high success rate for single object grasping.

#### 7.2.2. Multi-Object Sorting Experiment

**Figure 20:**
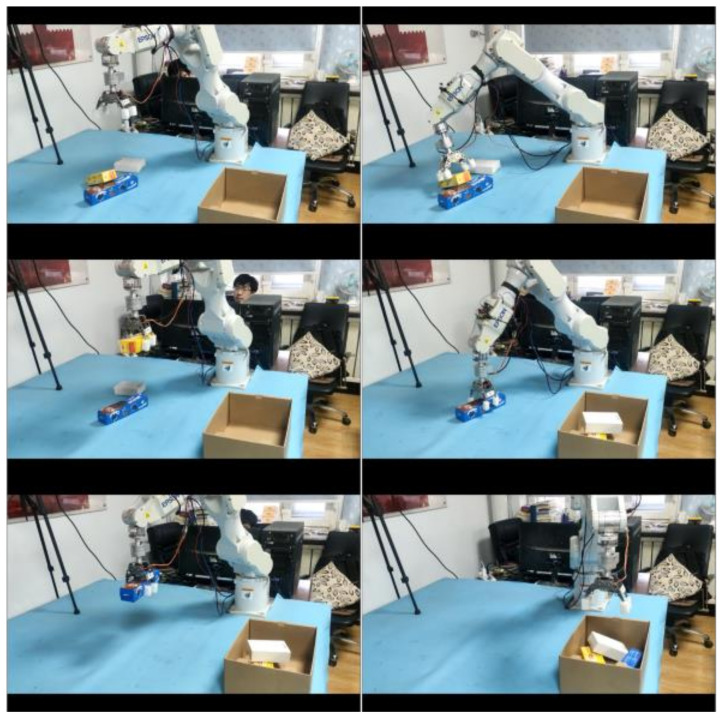
Multi-object sorting process.

The method proposed in this paper is not only applicable to the capture task of single-class objects but also to the sorting task of multiple-class objects. The specific sorting process is as follows: Firstly, different kinds of objects (2~4 per time) are stacked randomly on the experimental platform. The subsequent operation is described in Section 7.2.1. The difference is that the data transmitted from the Linux server not only contain the location information of the object, but also the category information of the object and the grab sequence information (obtained from Section 6.3). After starting sorting task, the robot will sort all the objects on the desktop orderly and move the objects to the specified location. The grasping process is shown in Figure 20.

The multi-object sorting experiments were conducted on two, three and four randomly placed target objects in the scene. Each experiment was conducted 200 times. In the experiment of two objects, the average number of grabs per object is 2×200÷5=80, and in the whole experiment, each object was grasped and moved 360 (80 + 120 + 160) times.

The multi-object sorting experiment is considered successful when all objects are captured in a reasonable order without causing other objects to fall, and experimental results of multi-object sorting are shown in Table 5. If a, b, c, m and t are set to represent apple, beer can, cookie box, mouse box and tea box, respectively, the grasping results and sorting order reasoning results in the multi-object sorting experiment are shown in Table 6 and Table 7, respectively. In Table 6, we use different numbers of repetitions for different amounts of objects, because we want to make the average number of times each object is captured in all experiments equal while ensuring that each type of experiment carries out the same number of experiments. The sign of successful capture in Table 6 is that the robot successfully captures the object and puts it in the correct pre-determined position without accidental collision during the process; otherwise, it is the capture failure. In Table 6, the success rates of grasping objects a, b, c, m and t are 91.67%, 100%, 100%, 100%, 100% and 97.22%, respectively. The success of sorting sequence reasoning in Table 7 is marked by the fact that the robot first grabs the object above in a stacked scene without affecting the location of other objects; otherwise, sorting order reasoning fails. The data results in Table 7 show that the sorting order reasoning algorithm proposed in this paper is reliable and can accurately predict the sorting order of multiple objects.

It can be seen from Table 4 and Table 6 that the grasping of apples is the most likely to fail, because the normal vector estimation of complex shape objects such as apples is not stable enough in this paper, so there is a certain deviation between the estimated grasping pose and the actual pose. In addition, it can be seen that the sorting success rate of multi-class objects is relatively lower than that of single-class objects, because the neural network constructed in this paper sometimes has the problem of missing detection in the case of more objects. However, it can be seen from Table 4 and Table 6 that the overall capture success rate and sorting success rate of the system constructed in this paper are high.

## 8. Conclusions and Future Work

In this paper, a robot multi-objective sorting system based on fusion of rotating target and instance segmentation network is constructed. The first part of the system is the object recognition system. The Kinect camera collects the images of objects in unstructured scenes and sends them into the trained rotation target detection network and the optimized instance segmentation network to obtain the pose and category information of objects. The second part is the robot grasping system. The Windows client sends the pose information of the object to the robot instruction after calculation and processing and drives the robot to grasp. This paper innovatively proposed to use the information of the upper surface of the object to obtain the actual pose of the object and determine the stacking relationship between the upper and lower objects by comparing the mean value of the upper surface point cloud in the z direction, so as to solve the sorting problem of stacked objects in the non-institutional scene. Real experimental results showed that the proposed method had very good performance and good robustness for the sorting of common objects in daily life.

In addition, the method proposed in this paper still has many places that can be further studied. Firstly, in this paper, the way to obtain the pose of the object is to extract the upper surface information. In the future, the recognition of the grab point or the extraction of the overall geometric characteristics of the object can be studied to obtain the pose of the object. In this paper, the method of combining two neural networks is used in object recognition system, and the two networks can be fused into a neural network in the future. In this paper, the obstacle avoidance problem is not considered in the process of robot grasping, and the system can be optimized by integrating obstacle avoidance factors. Finally, this paper does not consider the strength and stiffness of the objects in the grasping process and does not control the grasping forces for different situations. Research improvements in this area could be undertaken in the future.

## Figures and Tables

**Table 1:** Detection results of rotating target detection network.

IoU	Precision (%)	Recall (%)	F-Score (%)
0.5	0.992	0.988	0.990

**Table 2:** Dataset IOU calculation results (%).

Tea Box	Mouse Box	Cookie Box	Beer Can	Apple	MIoU
78.82	73.18	77.16	70.49	67.94	73.52

**Table 3:** Threshold of point cloud variance on target surface(cm^2^).

Tea Box	Mouse Box	Cookie Box	Beer Can	Apple
0.3	0.2	0.5	1.6	1.7

**Table 4:** Experimental results of single object grasping.

Object	Number of Successes	Number of Failures	Rate of Success
Cookie box	200	0	100%
Tea box	198	2	99%
Mouse box	199	1	99.5%
Apple	189	8	94.5%
Beer can	196	4	98%

**Table 5:** Threshold of point cloud variance on target surface(cm^2^).

	Objects Number	2	3	4
Method of this paper	experimental times	200	200	200
	number of successes	200	193	172
	rate of success	100%	96.5%	86%

**Table 6:** The grasping results.

Objects Number	2					3					4				
Class	a	b	c	m	t	a	b	c	m	t	a	b	c	m	t
Experimental times	80	80	80	80	80	120	120	120	120	120	160	160	160	160	160
Number of failures	0	0	0	0	0	5	2	0	0	0	15	6	2	2	3

**Table 7:** Sorting order reasoning results.

Objects Number	2					3					4				
Class	a	b	c	m	t	a	b	c	m	t	a	b	c	m	t
Experimental times	80	80	80	80	80	120	120	120	120	120	160	160	160	160	160
Error frequency	0	0	0	0	0	0	0	0	0	0	0	0	0	0	0
Accuracy	100%					100%					100%				

## Data Availability

The data presented in this study are available on request from the corresponding author.
